# Conservative Approach to Type III Ulnar Club Hand: Case Report

**DOI:** 10.1055/s-0041-1735137

**Published:** 2021-10-01

**Authors:** Gabriel Chartuni Teixeira Cury, Leonardo Álaf Lucinda de Sá, Cler David Oliveira, Nathália Vieira Caires, Arnaldo Gonçalves de Jesus Filho, Bruno Gonçalves Schroder e Souza

**Affiliations:** 1 Faculdade de Ciências Médicas e da Saúde de Juiz de Fora, MG, Brasil; 2 Departamento de Ortopedia e Cirurgia da Mão, Hospital Universitário, Universidade Federal de Juiz de Fora, MG, Brasil; 3 Departamento de Ortopedia e Cirurgia da Mão, Santa Casa de Misericórdia de Juiz de Fora, MG, Brasil; 4 Universidade Federal de Juiz de Fora, MG, Brasil

**Keywords:** hand deformities, congenital, musculoskeletal abnormalities, phocomelia, ulna/abnormalities, upper extremity deformities, congenital

## Abstract

Ulnar club hand is a rare condition of the upper limbs, for which treatment depends on the degree of morphological and functional impairment, correlating with the radiographic classification of Dobyns, Wood, and Bayne. The aim of the present study is to report a case of a 6-year-old male patient, followed up for type III ulnar club hand (total ulnar dysplasia). Despite the initial difficulty of manipulating objects and performing everyday tasks, conservative physical therapy treatment provided strength gain and development of functional skills for daily life. We conclude that patients with type III deformity can be properly managed with rehabilitation although they require outpatient follow-up until skeletal maturity is reached, as dynamic deformities and new functional limitations may lead to need for corrective surgeries.

## Introduction

Ulnar club hand (UCH) is a rare congenital condition. 1 In Brazil, an incidence of 1.6% of UCH was reported. 2 This is one of the rarest congenital deformities of the upper limbs. 2

Ulnar club hand is a longitudinal failure in the formation of the ulna (phocomelia), which may be totally or partially absent. 3 It is more frequent in men, on the right side, and unilateral in 70% of cases. The incomplete form is the most common. 4

The deformity is characterized by the forearm being short and curved to the radial side, with the hand diverted in the ulnar direction. 4 Other associated abnormalities may be present. 4 The elbow function may be compromised to different degrees (from stiffness to increased mobility, with instability). 1 5 In older children, dislocation of the radial head may occur. 1

Dobyns, Wood, and Bayne rank this condition in four types. In type I, there is ulna hypoplasia, small ulnar deviation, radio arching, and no anlage. In type II, there is partial dysplasia of the ulna (middle or distal third), the proximal joint of the ulna with the humerus is present, there is distal anlage and dislocation of the head of the radius. In type III, total dysplasia of the ulna occurs, there is no anlage, the radio is arched, and the head of the radio is dislocated (with elbow instability). In type IV, there is radio-humeral synostosis and anlage is usually present. 6

The objective of the present study is to present a case of type III UCH, in which the challenge was the choice of treatment. We discuss the elements that guide the therapeutic indication, those related to clinical follow-up, and present the functional results after 2 years of follow-up.

## Case Report

A 4-year-old male patient was admitted to a specialized outpatient clinic due to malformation of the right upper limb (RUL). The diagnosis of UCH had been performed by morphological ultrasound and confirmed after birth. In addition to UCH, left testicular agenesis was diagnosed. There was a family history of type III spinal muscular atrophy (paternal). This study was approved by the CEP (CAAE:97048518.9.0000.5103).

**Fig. 1:**
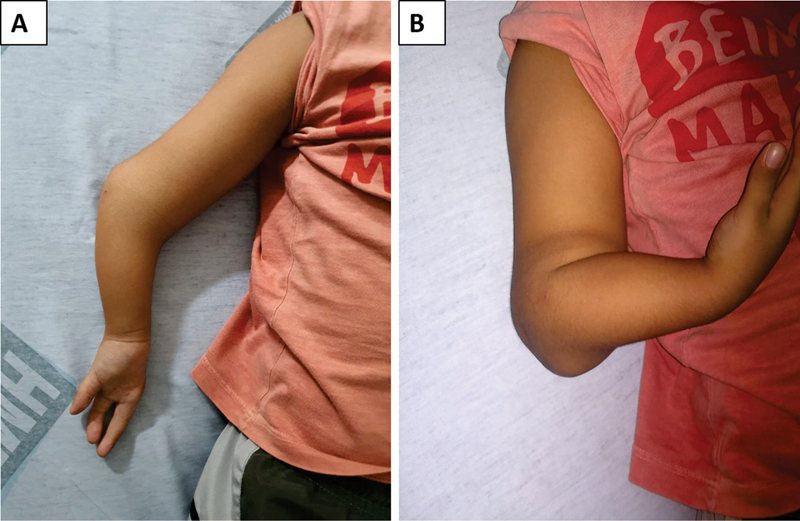
Ectoscopy of the deformity. (A) Anterior face of the member. (B) Profile with bending.

A typical deformity of UCH associated with agenesis of the fourth and fifth chirodactyls was evident ( Fig. 1 ). It was not possible to identify formation of fibrous or cartilaginous bar *(anlage).* There was important functional limitation and difficulties in manipulating objects as well as hypoesthesia at the medial edge of the third finger.

**Fig. 2:**
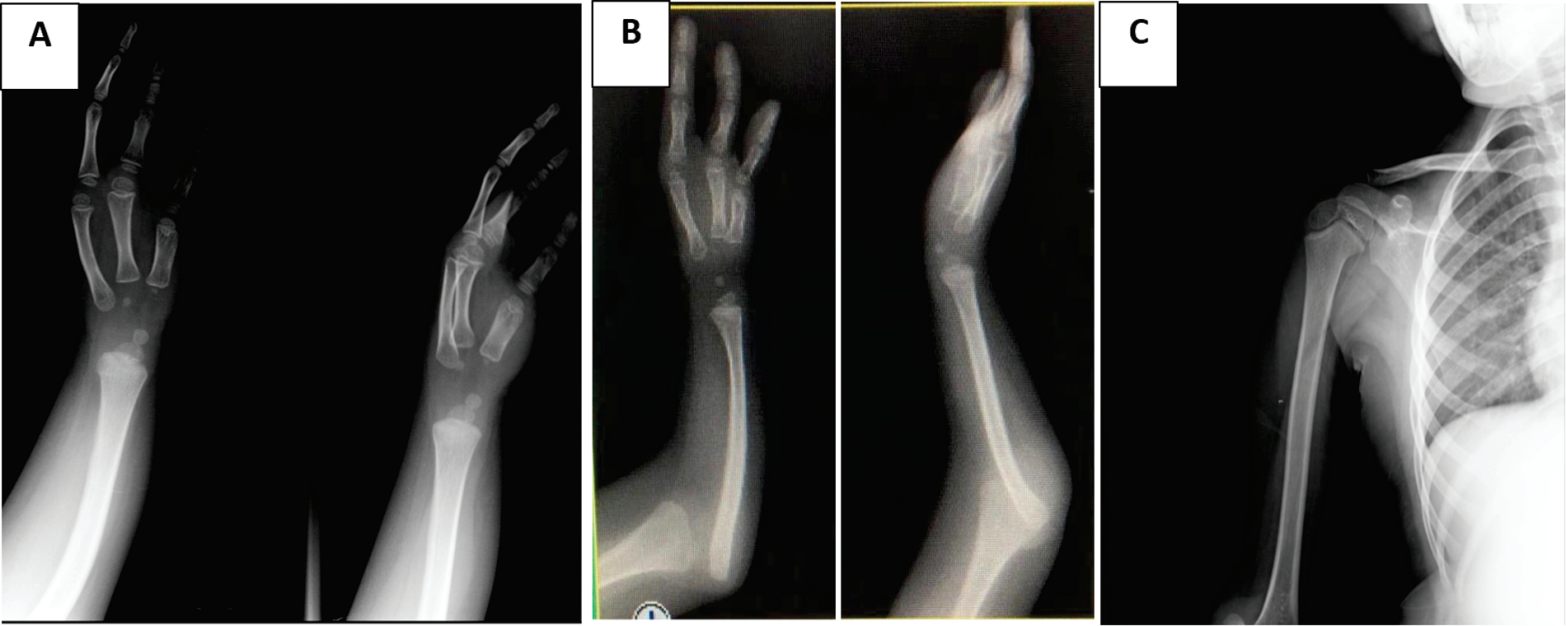
(A) Ulnar club hand type III of Dobyns, Wood, and Bayne – Total dysplasia of the ulna and absence of 4th and 5th right chirodactyls, and their respective metacarpals with preserved joint relationship. (B) Dislocation of the radio head. (C) Absence of deformities of the proximal humerus.

Radiographs ( Fig. 2 ) showed complete absence of ulna, dislocation of the head of the radio and centralized carpal bones. There was no glenohumeral dysplasia on the shoulder.

Given the limitations for daily life, the patient visited our outpatient clinic, where he received indication for physical therapy treatment. The goal was to optimize fine motor activity and functional gain (such as using the cell phone, getting dressed independently and putting on shoes). The treatment was performed for 9 months (1 hour, 3 times a week) ( Chart 1 ).

**Chart 1:** Physiotherapeutic procedures performed by the patient during the physiotherapy period

1. Strengthening of the right upper limb using a 0.5 kg shin force associated with the ball throwing activity, using an elastic band;
2. Strengthening of the phalanges of the right radiocarpic joint using wax mass, exercises for fingers and hands and rubber ball;
3. Strengthening of periscapular muscles associated with playful activities;
4. Joint mobilization of the scapular and glenohumeral;
5. Osteokinematic mobilization of the glenohumeral with assisting scapular;
6. Motor coordination training (learning to use phalanges individually);
7. Training function (tie shoelace, get dressed, open bottle).

The result of physical therapy was measured by the goal attainment scale (GAS) 7 , which included three tasks agreed on by family members and therapists. The objectives were: to improve the functionality of the RUL with gain of fine movements; to improve the palmar grip; to reduce scapular dyskinesia during abduction to pick up a toy on the shelf. At 2 months, the patient showed improvement in 2 tasks (video-compared RUL function and improvement of palmar grip in daily tasks).

In the consultation after physical therapy, we applied the disabilities of the arm, shoulder and hand (DASH) questionnaire. 8 As the patient was a child, questions 7, 8, 12 and 21 could not be applied because they addressed typical adult functions. This modification led us to consider not the absolute score (38.33 points), but its weighted value (46%), classifying the dysfunction as moderate. Chart 2 lists other findings.

**Chart 2:** Activity assessment before and after physiotherapy

	Before physical therapy	After physical therapy
**Function**	Limitation of the use of individual phalanx in the right hand Palmar grip limitation for performing functional tasks Scapular dyskinesia during abduction to pick up object on shelf	Acquisition of the ability to use individual phalanx (muscle strength: grade 4/5) Palmar grip enhancement No improvement observed
**Strength**	Right upper limb hypotonia Patient could move through full range of motion against gravity Patient could not carry weighted object/toy with right upper limb	Maintained Maintained Maintained
**Specific tasks**	Patient could feed himself Patient could use cell phone Patient could not get dressed independently Patient could not put his shoes on Patient could not open locks Patient could not open bottles Patient could not open packet of cookies	Maintained Improved with use of individual phalanx Partial function acquisition * Partial function acquisition Function acquisition ** Function acquisition Function acquisition

* Partial acquisition of function: Gain of function with difficulty and/or limitation in the execution of the movement.

** Function acquisition: Function gain with adequate adaptation to movement execution.

**Fig. 3:**
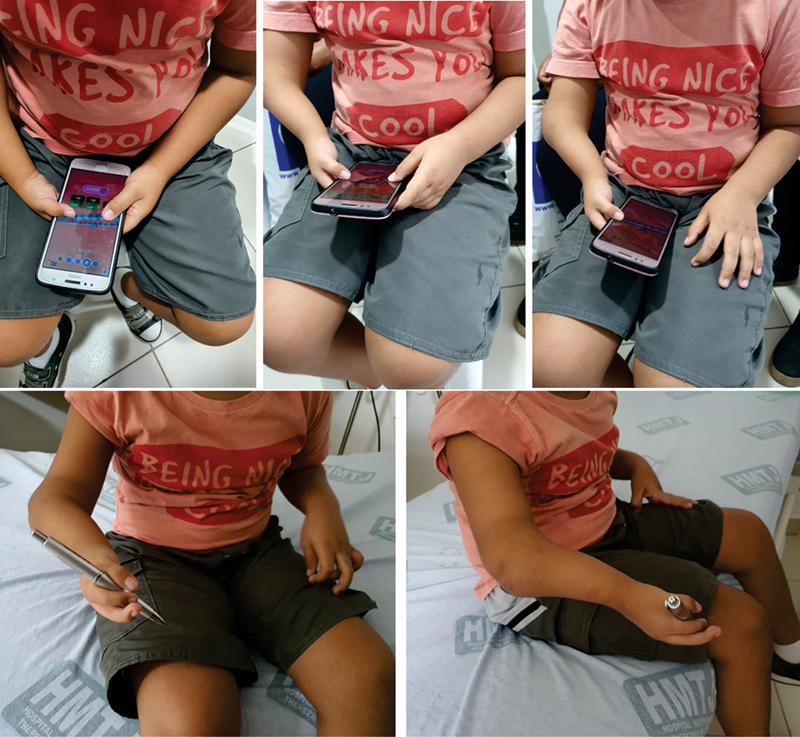
Illustrative images of limb functionality using electronic device and demonstrating clamp function.

The patient maintains follow-up after 2 years, asymptomatic and with stable condition, performs the movement of thumb tweezers, despite not presenting function of the opponent muscles. The flexor musculature of the three fingers is normal, o the patient is able to play with the cell phone, dress independently and perform other daily tasks ( Fig. 3 ). We chose not to indicate surgery at this time, although the approach can be reviewed, especially if there is loss of thumb tweezer ability, worsening of elbow stability, progressive loss of function, or pain.

## Discussion

Our case is illustrative of UCH because it includes a male patient and involvement of the right side. 4 In the literature, partial ulna agenesis (Bayne II) is the most frequent phenotype, which is different from our case. 1 5 6 We did not identify cases similar to ours in the national literature.

The agenesis of metacarpals and phalanges observed is also reported in most studies. 1 5 We did not identify concomitant musculoskeletal abnormalities or limitation of elbow mobility, unlike other authors. 5 6

The treatment in cases of UCH requires a multi-professional and individualized approach. 9 Most children are treated without surgery, especially in unilateral UCH. 9 In cases requiring surgery, the goal is to improve the function of the affected limb. 9

To evaluate the gain of function, we can use objective scales (e.g. DASH) or other more subjective scales (e.g.: GAS). Although validated, the DASH questionnaire has disadvantages because it involves a longer period of application and is specific to adults. Therefore, GAS is preferred by us, because it is more practical. It involves defining a set of objectives and specifies a range of possible outcomes for each objective (on a scale that contains 5 levels, from -2 to +2). It is used to evaluate performance after a specified intervention period. 7

The surgical approach in the cases of Bayne II was advocated by Monteiro and Schachinger. 1 5 In patients with Bayne IV, conservative treatment is recommended. 10 We did not identify recommendations for Bayne III. In this case, conservative treatment was chosen due to the patient's age and good response to physiotherapy with adequate adaptation to day-to-day activities.

The prognosis of UCH will depend on factors such as: unilateralism, association with syndromes, the number of fingers present, the degree of atrophy of the limbs, and the presence of sindactyly. 9 In our case, the function of the elbow and wrist joints may worsen with growth, which justifies serial follow-ups and eventual interventions. 1 However, the follow-up of the last 2 years showed satisfactory developments.
