# Supplementary material for: Conservative Approach to Type III Ulnar Club Hand: Case Report
Source: Rev Bras Ortop (Sao Paulo). 2021 Oct 1;59(Suppl 1):e12–6. doi: 10.1055/s-0041-1735137 (PMC11254420; doi:10.1055/s-0041-1735137)
Supplement: Related texts

# Research article: Abordagem conservadora da mão torta ulnar tipo III: Relato de caso

**Keywords:** anormalidades musculoesqueléticas, deformidades congênitas da mão, deformidades congênitas das extremidades superiores, focomelia, ulna/anormalidades

## Abstract

A mão torta ulnar é uma rara afecção de membros superiores, cujo tratamento depende do grau de comprometimento morfológico e funcional, que se correlaciona com a classificação radiográfica de Dobyns, Wood e Bayne. O objetivo deste estudo é relatar um caso de um paciente de 6 anos de idade, do sexo masculino, acompanhado devido a mão torta ulnar do tipo III (aplasia total da ulna). Apesar da dificuldade inicial de manipular objetos e realizar tarefas cotidianas, o tratamento conservador fisioterapêutico propiciou o ganho de força e o desenvolvimento de habilidades funcionais para a vida diária. Concluímos que pacientes com deformidade do tipo III podem ser manejados adequadamente com reabilitação embora necessitem de seguimento ambulatorial até a maturidade esquelética, pois deformidades dinâmicas e novas limitações funcionais podem levar à necessidade de cirurgias corretivas.

## Introdução

A mão torta ulnar (MTU) é uma afecção congênita rara. 1 No Brasil, uma incidência de 1,6% de MTU foi relatada. 2 Trata-se de uma das mais raras deformidades congênitas dos membros superiores. 2

A MTU é uma falha longitudinal na formação da ulna (focomelia), que pode estar total ou parcialmente ausente. 3 Ela é mais frequente em homens, no lado direito, e unilateral em 70% dos casos. A forma incompleta é a mais comum. 4

A deformidade é caracterizada por antebraço curto e curvado para o lado radial, com a mão desviada em sentido ulnar. 4 Outras anormalidades associadas podem estar presentes. 4 A função do cotovelo pode estar comprometida em diferentes graus (desde rigidez até aumento da mobilidade, com instabilidade). 1 5 Em crianças maiores, a luxação da cabeça radial poderá ocorrer. 1

Dobyns, Wood e Bayne classificam esta afecção em quatro tipos. No tipo I, há hipoplasia da ulna, pequeno desvio ulnar, arqueamento do rádio, e não existe anlage. No tipo II, há aplasia parcial da ulna (terço médio ou distal), a articulação proximal da ulna com o úmero está presente, existe anlage distal, e há luxação da cabeça do rádio. No tipo III, ocorre a aplasia total da ulna, não existe anlage, o rádio é arqueado, e a cabeça do rádio é luxada (com instabilidade do cotovelo). No tipo IV, há sinostose radioumeral e o anlage costuma estar presente. 6

O objetivo deste estudo é apresentar um caso de MTU tipo III, no qual o desafio foi a escolha do tratamento. Discutimos os elementos que norteiam a indicação terapêutica, os relacionados ao acompanhamento clínico e apresentamos os resultados funcionais após 2 anos de seguimento.

## Relato de caso

Um paciente de 4 anos de idade, do sexo masculino, foi admitido em ambulatório especializado devido a “má formação do membro superior direito (MSD)”. O diagnóstico de MTU havia sido realizado por ultrassonografia morfológica e confirmado após o nascimento. Além da MTU, foi diagnosticada agenesia testicular à esquerda. Havia história familiar de atrofia muscular espinhal tipo III (paterna). Este trabalho foi aprovado pelo CEP (CAAE:97048518.9.0000.5103).

Uma deformidade típica de MTU associada à agenesia do quarto e quinto quirodáctilos era evidente ( Fig. 1 ). Não foi possível identificar formação de barra fibrosa ou cartilaginosa (anlage). Havia importante limitação funcional e dificuldades para manipular objetos além de hipoestesia na borda medial do terceiro dedo.

**Fig. 1:** Ectoscopia da deformidade. (A) Face anterior do membro. (B) Perfil com flexão.

As radiografias ( Fig. 2 ) evidenciaram ausência completa da ulna, luxação da cabeça do rádio, e ossos do carpo centralizados. Não havia displasia glenoumeral no ombro.

**Fig. 2:** (A) Mão torta ulnar tipo III de Dobyns, Wood e Bayne – Aplasia total da ulna e ausência de 4° e 5° quirodáctilos direitos e seus respectivos metacarpos com relação articular preservada. (B) Luxação da cabeça do rádio. (C) Ausência de deformidades do úmero proximal.

Diante das limitações para a vida diária, o paciente procurou nosso ambulatório onde recebeu indicação de tratamento fisioterápico. O objetivo era otimizar a atividade motora fina e ganho funcional (como usar o celular, se vestir sozinho e colocar os sapatos). O tratamento foi realizado durante 9 meses (1 hora, 3 vezes por semana) ( Quadro 1 ).

**Quadro 1:** Procedimentos fisioterapêuticos realizados pelo paciente durante o período de fisioterapia

1. Fortalecimento de membro superior direito utilizando caneleira de 0,5 kg associado à atividade de arremessar bola, com uso de faixa elástica;
2. Fortalecimento das falanges da articulação radiocárpica direita utilizando massa de cera, exercitador para dedos e mãos e bola de borracha;
3. Fortalecimento dos músculos periescapulares associado a atividades lúdicas;
4. Mobilização articular da escapula e glenoumeral;
5. Mobilização osteocinemática da glenoumeral com assistência à escapula;
6. Treino de coordenação motora (aprender a utilizar as falanges de forma individual);
7. Treino de função (amarrar cadarço, vestir roupas, abrir garrafa).

O resultado da fisioterapia foi mensurado pela escala *Goal Attainment Scale* (GAS), 7 que elencou três tarefas consensuais entre os familiares e o terapeuta. Os objetivos foram: melhorar a funcionalidade do MSD com ganho de movimentos finos; aprimorar a preensão palmar; reduzir a discinesia escapular durante a abdução para pegar brinquedo na prateleira. Em 2 meses, o paciente apresentou melhora em 2 tarefas (função do MSD, comparada em vídeo, e melhora da preensão palmar nas tarefas diárias).

Na consulta após a fisioterapia, aplicamos o questionário *Disabilities of the Arm, Shoulder and Hand* (DASH). 8 Por ser criança, as questões 7, 8, 12 e 21 não puderam ser aplicadas por abordarem funções típicas dos adultos. Esta modificação nos levou a considerar não o escore absoluto (38,33 pontos), mas sim o seu valor ponderado (46%), classificando a disfunção como moderada. O Quadro 2 elenca outros achados.

**Quadro 2:** Avaliação de atividade antes e após a fisioterapia

	Antes da fisioterapia	Após a fisioterapia
**Função**	Limitação do uso de falange individual em mão direita Limitação da preensão palmar para realização de tarefas funcionais Discinesia escapular durante abdução para pegar objeto em prateleira	Aquisição da capacidade de usar falange individual (força muscular: grau 4/5) Aprimoramento da preensão palmar Sem melhora observada
**Força**	Hipotonia do membro superior direito O paciente conseguia vencer gravidade O paciente não conseguia carregar objeto/brinquedo com peso com membro superior direito	Mantido Mantido Mantido
**Tarefas específicas**	O paciente conseguia se alimentar sozinho O paciente conseguia usar o celular O paciente não conseguia se vestir sozinho O paciente não conseguia colocar os sapatos O paciente não conseguia abrir fechaduras O paciente não conseguia abrir garrafas O paciente não conseguia abrir pacote de biscoitos	Mantido Melhora com uso de falange individual Aquisição parcial de função ** Aquisição parcial de função Aquisição de função ** Aquisição de função Aquisição de função

* Aquisição parcial de função: Ganho de função com dificuldade e/ou limitação na execução do movimento.

** Aquisição de função: Ganho de função com adequada adaptação à execução do movimento.

O paciente mantém o acompanhamento após 2 anos, assintomático e com o quadro estável, realiza o movimento de pinça do polegar, apesar de não apresentar função da musculatura de oponência. A musculatura flexora dos três dedos é normal, então o paciente é capaz de brincar com o celular, se vestir independentemente e realizar outras tarefas diárias ( Fig. 3 ). Optamos por não indicar cirurgia neste momento, embora a conduta possa ser revista, principalmente se houver perda da capacidade de pinça do polegar, piora da estabilidade do cotovelo, perda progressiva da função, ou dor.

**Fig. 3:** Imagens ilustrativas da funcionalidade do membro utilizando aparelho eletrônico e demonstrando função de pinça.

## Discussão

Nosso caso é ilustrativo da MTU, pois inclui paciente do sexo masculino, de acometimento no lado direito. 4 Na literatura, a agenesia parcial da ulna (Bayne II) é o fenótipo mais frequente, diferente do nosso caso. 1 5 6 Não identificamos casos semelhantes ao nosso na literatura nacional.

A agenesia de metacarpos e falanges observada também é reportada na maioria dos estudos. 1 5 Não identificamos anormalidades musculoesqueléticas concomitantes ou limitação da mobilidade do cotovelo, ao contrário de outros autores. 5 6

O tratamento nos casos de MTU requer abordagem multiprofissional e individualizada. 9 A maioria das crianças é tratada sem cirurgia, especialmente na MTU unilateral. 9 Nos casos que necessitam de cirurgia, o objetivo é melhorar a função do membro afetado. 9

Para avaliar o ganho de função podemos utilizar escalas objetivas (ex.: DASH) ou outras mais subjetivas (ex.: GAS). Embora seja validado, o questionário DASH apresenta desvantagens por envolver um período maior de aplicação e ser específico para adultos. Por isso, o GAS é preferido por nós, por ser mais prático. Ele envolve definir um conjunto de objetivos, especificando uma gama de possíveis resultados para cada objetivo (em uma escala que contém 5 níveis, de -2 a +2). É utilizado para avaliar o desempenho após um período de intervenção especificado. 7

A abordagem cirúrgica nos casos de Bayne II foi advogada por Monteiro e Schachinger. 1 5 Em pacientes com Bayne IV, o tratamento conservador é recomendado. 10 Não identificamos recomendações para Bayne III. Neste caso, optou-se pelo tratamento conservador devido à idade do paciente e boa resposta à fisioterapia com adaptação adequada às atividades do dia a dia.

O prognóstico da MTU dependerá de fatores como: unilateralismo, associação a síndromes, o número de dedos presentes, o grau de atrofia dos membros e a presença de sindactilia. 9 Em nosso caso, a função de articulações do cotovelo e punho pode piorar com o crescimento, o que justifica acompanhamentos seriados e eventuais intervenções. 1 No entanto, o seguimento dos últimos 2 anos evidenciou evolução satisfatória.
